# An integrated view of correlated emissions of greenhouse gases and air pollutants in China

**DOI:** 10.1186/s13021-023-00229-x

**Published:** 2023-05-19

**Authors:** Xiaohui Lin, Ruqi Yang, Wen Zhang, Ning Zeng, Yu Zhao, Guocheng Wang, Tingting Li, Qixiang Cai

**Affiliations:** 1 State Key Laboratory of Atmospheric Boundary Layer Physics and Atmospheric Chemistry, Institute of Atmospheric Physics, Chinese Academy of Sciences, Beijing, China; 2 State Key Laboratory of Numerical Modeling for Atmospheric Sciences and Geophysical Fluid Dynamics, Institute of Atmospheric Physics, Chinese Academy of Sciences, Beijing, China; 3 Department of Atmospheric and Oceanic Science, and Earth System Science Interdisciplinary Center, University of Maryland, College Park, Maryland USA; 4 State Key Laboratory of Pollution Control & Resource Reuse and School of the Environment, Nanjing University, 163 Xianlin Ave, Nanjing, Jiangsu China; 5 Southern Marine Science and Engineering Guangdong Laboratory (Zhuhai), Zhuhai, Guangdong China

**Keywords:** Greenhouse gases, Air pollutants, Correlation, Policy making, Synergistic emissions reduction

## Abstract

**Background:**

Air pollution in China has raised great concerns due to its adverse effects on air quality, human health, and climate. Emissions of air pollutants (APs) are inherently linked with CO_2_ emissions through fossil-energy consumption. Knowledge of the characteristics of APs and CO_2_ emissions and their relationships is fundamentally important in the pursuit of co-benefits in addressing air quality and climate issues in China. However, the linkages and interactions between APs and CO_2_ in China are not well understood.

**Results:**

Here, we conducted an ensemble study of six bottom-up inventories to identify the underlying drivers of APs and CO_2_ emissions growth and to explore their linkages in China. The results showed that, during 1980–2015, the power and industry sectors contributed 61–79% to China’s overall emissions of CO_2_, NO_x_, and SO_2_. In addition, the residential and industrial sectors were large emitters (77–85%) of PM_10_, PM_2.5_, CO, BC, and OC. The emissions of CH_4_, N_2_O and NH_3_ were dominated by the agriculture sector (46–82%) during 1980–2015, while the share of CH_4_ emissions in the energy sector increased since 2010. During 1980–2015, APs and greenhouse gases (GHGs) emissions from residential sources generally decreased over time, while the transportation sector increased its impact on recent emissions, particularly for NO_x_ and NMVOC. Since implementation of stringent pollution control measures and accompanying technological improvements in 2013, China has effectively limited pollution emissions (e.g., growth rates of –10% per year for PM and –20% for SO_2_) and slowed down the increasing trend of carbon emissions from the power and industrial sectors. We also found that areas with high emissions of CO, NO_x_, NMVOC, and SO_2_ also emitted large amounts of CO_2_, which demonstrates the possible common sources of APs and GHGs. Moreover, we found significant correlations between CO_2_ and APs (e.g., NO_x_, CO, SO_2_, and PM) emissions in the top 5% high-emitting grid cells, with more than 60% common grid cells during 2010–2015.

**Conclusions:**

We found significant correlation in spatial and temporal aspects for CO_2_, and NO_x_, CO, SO_2_, and PM emissions in China. We targeted sectorial and spatial APs and GHGs emission hot-spots, which help for management and policy-making of collaborative reductions of them. This comprehensive analysis over 6 datasets improves our understanding of APs and GHGs emissions in China during the period of rapid industrialization from 1980 to 2015. This study helps elucidate the linkages between APs and CO_2_ from an integrated perspective, and provides insights for future synergistic emissions reduction.

**Supplementary Information:**

The online version contains supplementary material available at 10.1186/s13021-023-00229-x.

## Background

Air pollution has raised great concerns in regard to climate [1, 2], air quality [3], and human health [4, 5]. As a large emitter of greenhouse gases (GHGs), China is also facing a great pressure due to environmental problems [6–8]. The Chinese government has issued guidelines to coordinate the reduction of pollution and GHGs emissions to achieve the goal of carbon peak before 2030 and carbon neutrality before 2060 [9]. Several air pollutants (APs), such as BC and ozone precursors tend to amplify warming by absorbing and scattering radiation and modifying cloud formation and optical properties [10–13]. APs emissions could thus influence the size of the CO_2_ budget to limit warming [14]. As APs and GHGs are often emitted by similar sources [15, 16], synergy between pollution and carbon emissions control measures is imperative. Moreover, the inclusion of air quality co-benefits in climate policies could yield notable implications for respiratory health and food security [17–19]. Knowledge of spatiotemporal variations and exploration of the possible linkages between APs and GHG are crucial first steps to effectively mitigate both air pollution and climate change.

An emissions inventory is generally developed to provide fundamental information to better understand the sources and trends of anthropogenic emissions. Gridded emissions are applied as inputs into Earth system models (ESMs) and chemistry transport models (CTMs). The accuracies of emissions inventories largely depend on their associated methodology and input data (Andres 2019). Alternatively, satellite data have been widely used to derive up-to-date anthropogenic emissions (e.g. SO_2_, NO_x_, and NH_3_) at the local, regional and global scales [20–23]. Satellite-based observations provide a good overview of the total amount of emissions, but remain limited in explaining emissions source contributions. Several efforts have been made to study the linkage between APs and GHGs and the potential synergy of emissions control [24–26]. Actions to mitigate APs can simultaneously reduce co-emitted CO_2_, thus generating co-benefits to accomplish climate change mitigation [27, 28]. Moreover, certain short-lived APs are strongly reduced by mitigation measures to limit CO_2_ emissions [14]. Concerns have been raised regarding integrated assessment of APs and GHG emissions originating from specific sources such as transport [29–31], waste [32, 33], cement industry [34, 35], and power plants [36, 37]. However, because of economic growth and air pollution regulations, the emissions characteristics of GHGs and source-related components change over time and by location [38, 39]. In addition, current available emissions inventories differ in species, magnitude of emissions estimates, spatiotemporal resolutions, spatial coverage, list of compounds and sector-specific details of source calculations [40]. Possible interactions among different species are not fully understood due to multiple source categories and physical/chemical processes [27, 30].

Anthropogenic emissions of GHGs and APs in China have undergone major increases over recent decades due to rapid economic growth, industrialization, and urbanization. In 2019, the major APs emissions in China contributed approximately 14%–28% to global emissions [41]. Moreover, the GHGs (mainly characterized as CO_2_, CH_4_, and N_2_O in this study) emissions in China contributed 15%–29% to global emissions [41]. China is facing the dual challenges of simultaneously reducing air pollution and carbon emissions [19, 42]. To improve the air quality, stringent air pollution control measures were implemented by the Chinese government after the State Council of China released the ‘Air Pollution Prevention and Control Action Plan’ in 2013 and the ‘Three-Year Action Plan to Win the Blue Sky Defense Battle’ in 2018. Since 2013, the growth trend of PM_2.5_ concentrations in China has been curbed by recent clean air measures, at an average decreasing rate ranging from 4.0 to 5.2 μgm^−3^ yr^−1^ [43, 44]. Except for particulate emissions, the effectiveness of clean air policies has also been observed in terms of multi-pollutants emissions and the source contributions, such as SO_2_ and NO_x_ emissions stemming from the industrial and power sectors [45, 46].

Previous studies have provided a general overview of APs and GHGs emissions and their variations across China [47–49] or in heavily polluted regions such as the Beijing-Tianjin-Hebei (BTH) region [50, 51], the Yangtze River Delta (YRD) region [52, 53], and Shanxi Province [54, 55]. However, the linkages and interactions between APs and GHGs at the national scale are not well characterized. Therefore, quantifying the characteristics and changes of APs and GHGs emissions and revealing their relationships at large spatiotemporal scales are fundamentally important to improve the air quality and realize climate change mitigation. Here, we conducted a comprehensive study, comprehensively considering APs and GHGs emissions to identify the differences of emissions and their spatial–temporal correlations and linkages. Based on the most comprehensive public emissions inventories, we presented a detailed evaluation of the major emission sources, including the agriculture, power, industrial, residential, transportation, and waste sectors. We also aim to characterize trends, sector-specific emissions and correlations of anthropogenic GHGs and APs emissions in China and provide scientific basis for addressing both air quality and climate problems more effectively from an integrated perspective.

## Methods

To explore the spatial patterns and trends of GHGs and APs emissions and their linkages in China, we analyzed six global and regional bottom-up inventories including 5 gridded datasets and 1 tabular dataset. The 5 gridded inventories considered in this study included the Community Emission Data System (CEDS v20210421) [41], Emissions Database for Global Atmospheric Research (EDGAR v5.0) [56], Multi-resolution Emission Inventory for China (MEIC v1.3) [43], Peking University (PKU v2) [57], and Regional Emissions Inventory in Asia (REAS v3.2) [58]. The statistical tabular dataset was retrieved from the published study of Zhao [59]. Specifically, CEDS v20210421 is a global annual emission inventory based on a mosaic approach that provides country-level emissions by fuel and sector of the GHGs (i.e., CO_2_, CH_4_, and N_2_O) and APs (e.g., CO, NO_x_, SO_2_, NH_3_, BC, OC, and NMVOC) during 1750–2019 [41]. EDGAR v5.0 was developed by the European Commission’s Joint Research Centre (JRC) and the Netherlands Environmental Assessment Agency (PBL), which provides sectoral and country-level emissions of GHGs (i.e., CO_2_, CH_4_, and N_2_O) and APs including ozone precursor gases (e.g., CO, NMVOC, and NO_x_), acidifying gases (e.g., NO_x_, and SO_2_), and primary particulates (e.g., PM_2.5_, PM_10_, BC, and OC) during 1970–2015 [60]. PKU v2 is a global monthly emission inventory that includes GHGs (i.e., CO_2_ and CH_4_) and APs (e.g., PM_2.5_, PM_10_, CO, NO_x_, SO_2_, TSP, NH_3_, BC, OC, PAHs) during 1960–2014 based on 64 to 88 (except CH_4_) individual sources [61, 62]. REAS v3.2 produces monthly Asian inventories of sector-specific emissions of CO_2_, PM_2.5_, PM_10_, CO, NO_x_, SO_2_, NH_3_, BC, OC and NMVOC during 1950–2015 [58]. In MEIC v1.3, a technology-based approach is implemented by Tsinghua University to produce monthly anthropogenic emissions inventories over mainland China for CO_2_, PM_2.5_, PM_10_, CO, NO_x_, SO_2_, NH_3_, BC, OC, and NMVOC in 2008 and 2010–2017 [45, 63]. In addition, we also used one statistical tabular dataset constructed by Zhao, Zhang (59), which contains a national-scale and sector-specific emissions for CO_2_ and pollutants (e.g. PM_2.5_, PM_10_, CO, NO_x_, SO_2_, BC, and OC) over the period of 2000–2014 in China [64]. To evaluate the characteristics of emissions under the different inventories on a common scale, specific anthropogenic sectors were aggregated into six categories (i.e., the agriculture, power, industrial, residential, transportation, and waste sectors). Further details on the inventories adopted in this study are listed in Table 1. The differences and uncertainties of different inventories were showed in Additional file 1: Fig. S5 and Table S1.

**Table 1:** Key features of the emissions inventories considered in this study

Item	PKU (PKU-FUEL)	CEDS (CEDS v2021-04–21)	EDGAR (EDGARv5.0)	MEIC (MEIC v1.3)	REAS (REAS v3.2)	Zhao et al., (2013)
Year	1960–2014	1950–2019	1970–2015	2008,2010–2017	1950–2015	2000–2014
Domain	Global	Global	Global	China	East, Southeast, South, and Central Asia	China
Spatial resolution	0.1	0.5	0.1	0.25	0.25	NA
Temporal resolution	Monthly	Monthly	Annual	Annual	Monthly	Annual
Data access	http://inventory.pku.edu.cn/home.html	http://www.globalchange.umd.edu/ceds/ceds-cmip6-data/	https://edgar.jrc.ec.europa.eu/overview.php?v=50_GHG	http://meicmodel.org/?page_id=560	http://www.nies.go.jp/REAS/index.html#data%20sets	Data developer
Reference	[57]	[41]	[60]	[43]	[58]	[59]

*NA* not applicable.

To quantify the spatial characteristics of CO_2_ and APs emissions, we conducted target analysis in seven high-emitting areas, including the Beijing-Tianjin-Hebei region and surrounding provinces (Henan and Shandong) (BTHs), the YRD region, the Pearl River Delta (PRD) region, the Cheng-Yu (CY) region, the Fenwei Plain (FP), Northeast China (NE), and the Triangle of Central China (TC). Moreover, the top 5% high-emitting grid cells derived from specific emissions were collected to explore the spatial linkages between carbon and pollutant emissions. Specifically, the spatial locations of the top 5% high-emitting grids for APs were identified and then compared to the exact locations of the top 5% CO_2_ high-emitting grids to identify common grids.

## Results and discussions

### Temporal variations of air pollution and greenhouse gases emissions in China

**Fig. 1:**
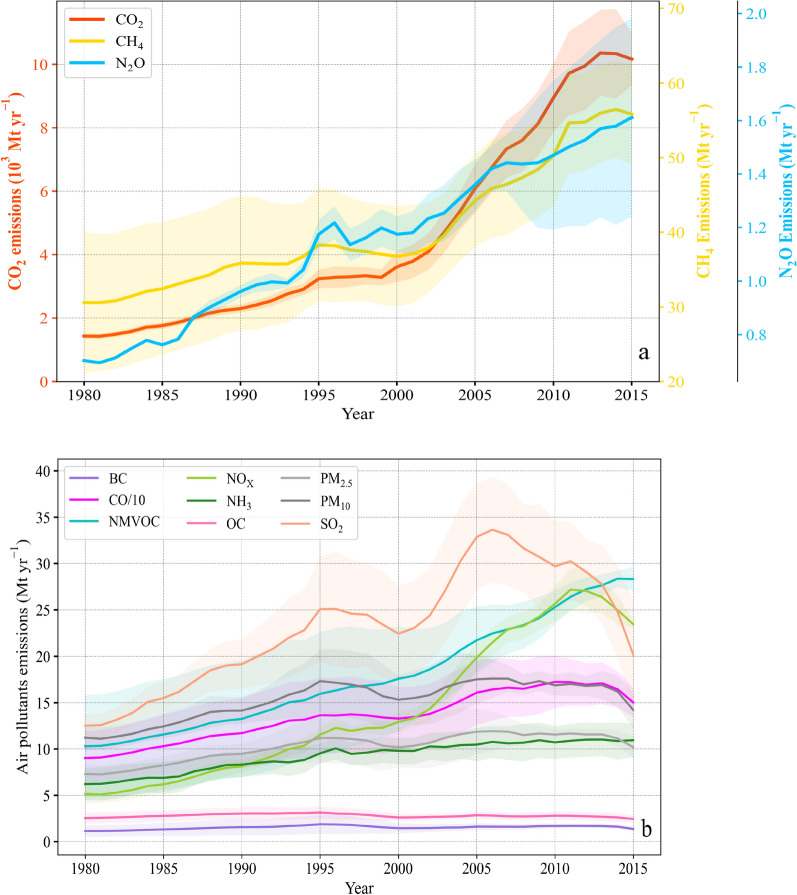
Temporal variations of the ensemble mean GHGs (**a**) and APs (**b**) emissions in China since 1980. The shaded areas represent one standard deviation of the six inventories used (i.e. PKU, CEDS, EDGAR, MEIC, REAS, Zhao et al. (2013)), and see Additional file 1: Table S1 and Fig. S5 for their differences and uncertainties

The ensemble mean anthropogenic GHG emissions in China exhibited high growth rates in the 2000s, and later, the increase rates declined during 2010–2015 (Fig. 1a). CO_2_ and CH_4_ emissions exhibited relatively low growth rates before 2000, with annual average growth rates (AAGRs) of 4.5% and 1.0%, respectively, and these rates increased notably during 2000–2009 (AAGRs: 9.4% and 3.1%, respectively), but thereafter gradually increased during 2010–2015 (AAGRs: 2.6% and 2.1%, respectively). Fossil fuel combustion, coal mining, and livestock are major drivers of the trends of CO_2_ and CH_4_ emissions in China [65–67]. Recent trends of emissions have stabilized or decreased mostly due to industrial structure updating, combustion efficiency improvement, and emissions control [67, 68]. CH_4_ emissions increased from 2010 to 2015. The main driving factor is the increased raw coal production. It increased from 2378.4 Mt to 2615.0 Mt (9.9% increase) during the period of 2010–2015 [69], which contributing 75% of primary energy production and thus driving the increased CH_4_ emissions [65]. The increased CH_4_ emissions from fossil production was also accompanied by their consumptions and thus the corelated increased fossil APs (e.g. NO_x_, SO_2_) emissions (Fig. 1), while the increase of agricultural CH_4_ was corelated with NH_3_ and NMVOC. CH_4_ emissions differ widely among existing inventories, and the emissions estimates for 1980 and 2015 range from 25.3 to 41.5 Mt CH_4_ yr^−1^ and 51.1–60.6 Mt CH_4_ yr^−1^, respectively. This result probably occurs because of the higher estimates in EDGAR obtained based on higher emission factors for rice cultivation and coal mining [65, 66]. Thus the use of these inventory should be careful and when focus on local areas, we recommend use inventories compiled from local activity data and emission factors (e.g. PKU and Zhao). N_2_O emissions continued to steadily increase, with an AAGR of 2.8% (before 2000) and the rate declined thereafter, with AAGRs of 2.3% and 1.9% in the 2000s and 2010–2015, respectively. Since the increase of N_2_O emissions is mainly driven by agricultural nitrogen fertilizer applications, it was positive correlated with NH_3_ emissions. However, a clear discrepancy was found in N_2_O emissions since 2007 among the inventories, which resulted from the continuing upward trend in CEDS (AAGR: 2.8%) and the downward trend in EDGAR (AAGR: −0.3%) during 2007–2015 (Additional file 1: Fig. S1). These results are attributed to the differences in source categories, input activity data, and emission factors between the currently available emissions inventories [40, 70]. The N_2_O emissions in the agriculture and energy sectors reported by the Food and Agriculture Organization (FAO), indicated a slightly increasing trend (AAGR: 0.8%) during 2007–2015. As the nationwide reduction in N-fertilizer applied per area has almost been offset by the expansion of the sowing area, the growth rate of N_2_O emissions stemming from croplands declined after 2003 and then plateaued until 2014 [71].

Regarding APs, the ensemble mean of NO_x_, NMVOC, and SO_2_ emissions revealed large variations, and CO, NH_3_, and PM emissions exhibited relatively small variations, but BC and OC indicated negligible variations during 1980–2015 (Fig. 1b). The notable increase in SO_2_ emissions was mainly caused by emissions originating from coal combustion in power plants in the 2000s, but considerable reductions subsequently occurred after 2006 due to the introduction of flue gas desulfurization (FGD) systems and the improvement in fuel combustion efficiency [18, 72]. The SO_2_ emissions exhibited a steep decreasing trend (AAGRs: −5.5%) during 2006–2015, with a peak value of 33.7 Mt in 2006 (Fig. 1b). Furthermore, SO_2_ emissions were estimated to continue to decrease (AAGR: −19.6% during 2013–2017, Additional file 1: Fig. S1) because of the shutdown of small coal-fired industrial boilers and the replacement of residential coal use with electricity and natural gas in recent years [45]. NO_x_ emissions increased rapidly in the 2000s (AAGR: 7.2%), but decreased from 2012 onward (AAGR: −4.6%, Fig. 1b). These results are attributed to the introduction of denitrification technology (selective catalytic reduction, SCR) in large power plants and regulations targeting road vehicles [58]. NMVOC emissions maintained an almost continuous increase trend, with an AAGR of 2.9% during 1980–2015 (Fig. 1b). This growth was mainly due to the persistent growth in emissions from the industrial sector and solvent use [70], with a lag in effective emissions controls in current policies [45]. PM, such as PM_2.5_ and PM_10_, exhibited a consistent trend with that of SO_2_, with peaks reached in approximately 2005. Later, particulate pollution decreased along with SO_2_ due to the installation of FGD systems in power plants. Since 2013, the implementation of clean air policies has led to considerable PM_2.5_ and PM_10_ emissions reductions (AAGRs: −9.5% and −12.1%, respectively) during 2013–2017, which are consistent with the results from previous studies [43, 63]. The implementation of stringent pollution control policies in China has effectively reduced growth rates, despite an increase in fossil fuel consumption and vehicle numbers [73, 74].

BC and OC contain relatively higher uncertainties among the APs because of the lack of sufficient information on the energy consumption, combustion technology, and emissions rate in the rural residential sector [51, 57, 75]. The differences among the various inventories range from 63 to 71% in 2015 (Fig. S2). In specific sectors, there are also considerable discrepancies in NO_x_, SO_2_ and PM emissions originating from industrial sources, and the differences among the current inventories range from 40% to 98% in 2015 (Additional file 1: Fig. S2). The uncertainties in NO_x_ and PM emissions are attributed to the emissions from cement production and industrial boilers [76]. CO emissions also indicate a large discrepancy in residential sources, and the estimates range from 29.4 to 67.1 Mt yr^−1^ in 2015. As evidenced by the substantial emissions of APs and GHGs in China and their consequences for climate change and public health, reliable inventories are of great importance in both understanding emission sources and supporting GHGs reduction and air quality improvement.

### Sectoral contributions to the changes in GHGs and APs emissions across China

**Fig. 2:**
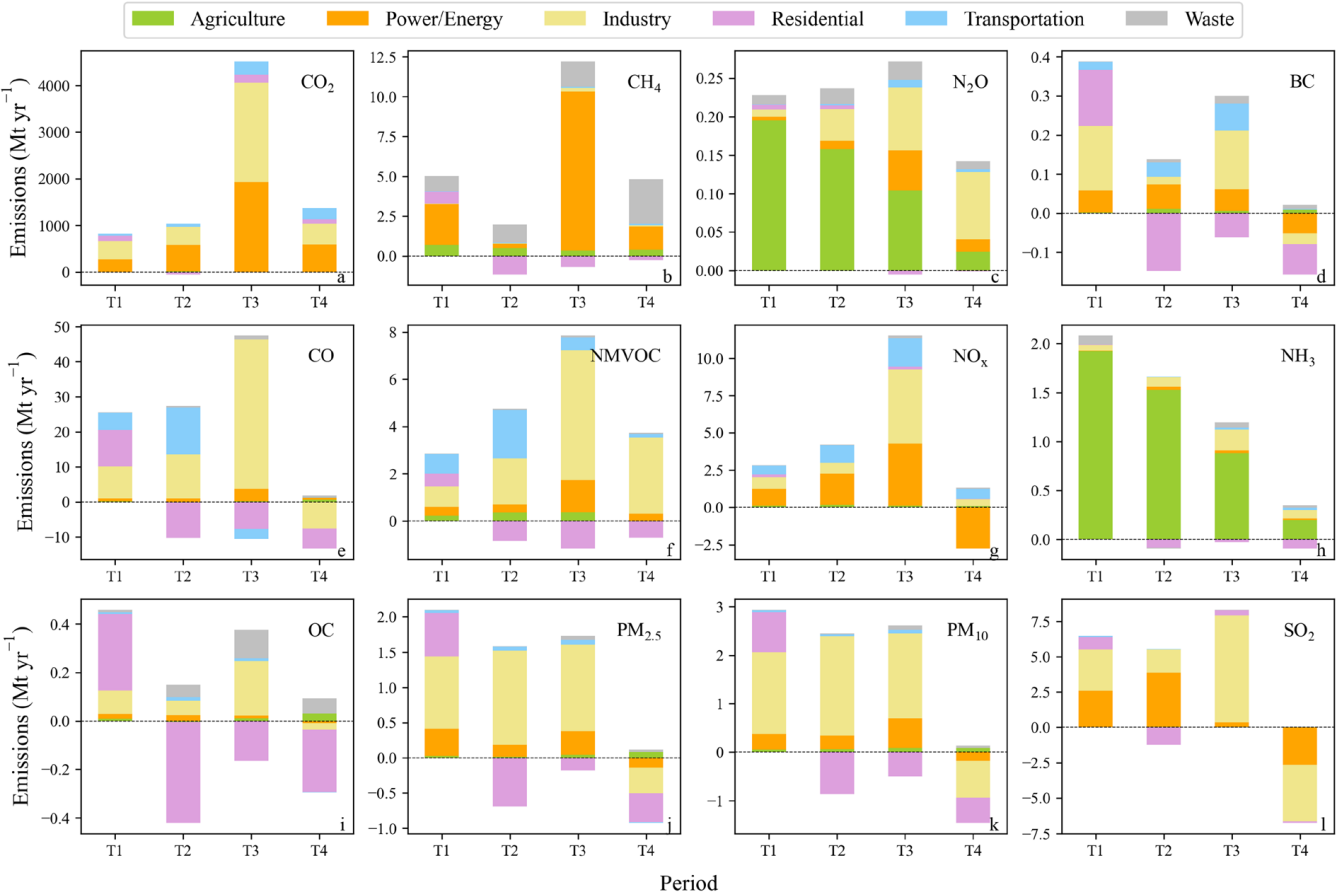
Sectoral contributions to the changes in GHGs and APs emissions across China in the 1980s (T1), 1990s (T2), 2000s (T3), and 2010–2015 (T4)

Quantification of the relative contributions of the different sectors and the evolution of high-emitting sources over time allowed us to target sector-specific emissions reductions. The temporal changes of the ensemble mean GHGs emissions across China in the 1980s (T1), 1990s (T2), 2000s (T3), and after 2010 (T4) were comprehensively determined based on the different inventories. Regarding the specific sources, the power and industry sectors played a dominant role in CO_2_ emissions growth, contributing 33.3%–57.1% to the increase during the different periods (Fig. 2a). CH_4_ emissions growth was mainly driven by the power sector (31.5%–86.7%), and the impact of the emissions of the waste sector increased after 2010 (Fig. 2b). CH_4_ emissions from coal production were curbed by closing a large number of small mines and increasing the efficiency in larger coal mines since 2010 [68]. In contrast, most of the N_2_O emissions originated from agricultural sources, while industrial sources increased their impact on recent emissions, accounting for 62.6% of the growth after 2010 (Fig. 2c).

In terms of specific sectors, the power and industrial sectors contributed the most (61–79%) to CO_2_, NO_x_, and SO_2_ emissions, while the residential and industrial sectors were large emitters (77–85%) of PM_10_, PM_2.5_, CO, BC, and OC during 1980–2015 (Additional file 1: Fig. S2). These results further confirmed that certain APs and CO_2_ are correlated, as they are all strongly associated with fossil-energy combustion [15]. CH_4_, N_2_O and NH_3_ were dominated by the agriculture sector (46–82%), while the energy sector increased its share of CH_4_ emissions after 2010 (Fig. 2b). The shares of both APs and GHGs emissions decreased in the residential sector, especially CO, BC, and NMVOC emissions, with the proportions decreasing by more than 30%. These results are probably attributed to the reduced emissions originating from biofuel combustion in recent years [58]. As the number of vehicles increased, the transportation sector increased its impact on recent emissions. For example, in terms of NO_x_ and NMVOC emissions, the transportation sector contributed 9% and 14%, respectively, to the total emissions in 1980, and the contribution rates later increased to 18% and 27%, respectively. However, APs emissions stemming from transportation increased less than did CO_2_ emissions because of vehicle technology improvement and fuel sulfur content reduction [30].

After the implementation of strict control measures, APs emissions began to decline or a negative growth was observed afterward. For example, the industrial sector contributed 84.9% to the decline in CO emissions during 2010–2015, which was beneficial for the improvement in the combustion efficiency and regulation tightening [77]. The power and industrial sectors contributed 45.8% and 51.5%, respectively, to SO_2_ emissions reduction since 2010 (Fig. 2l), which resulted from the phasing out of shaft kilns in cement production [78, 79] and from the introduction of ultra-low emissions standards for coal-fired power plants [74]. These results indicated that mitigation measures were the dominant factor contributing to pollution emissions reduction and reducing carbon emissions originating from the power and industrial sectors.

In regard to BC and OC, residential sources dominated the variation in total emissions, contributing more than 60% to emissions reduction since 2010 (Fig. 2d, i). NH_3_ emissions exhibited similar features to those of N_2_O emissions, with agriculture as the largest source, but its share gradually declined during 1980–2015. In terms of PM, PM_10_ and PM_2.5_ emissions increased steadily due to the growth in emissions in the industrial sector during 1980–2009 (Fig. 2j, k). Recently, PM emissions have experienced substantial changes since 2010, which can be attributed to the integrated effect of emissions reduction in the power, industrial, and residential sectors. The reduction in emissions of certain APs could also lead to a decrease trend in PM emissions during 2010–2015. For example, BC is a major component of primary PM_2.5_, whereas SO_2_ and NO_x_ (precursors of sulfate and nitrate aerosols, respectively) are crucial precursors in the formation of secondary PM_2.5_ [58].

### Spatial relationships between CO_2_ and APs emissions in high-emitting areas

**Fig. 3:**
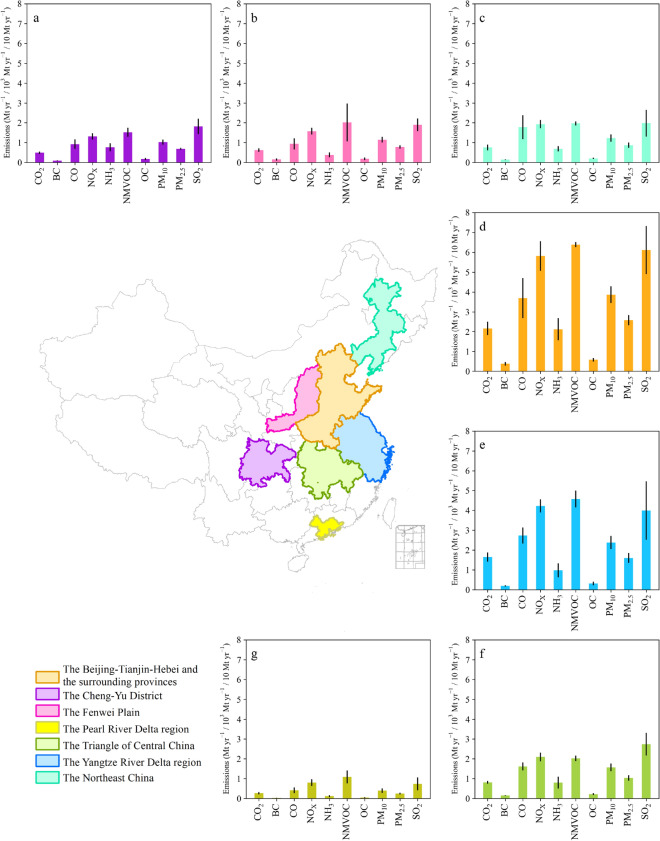
The high-emitting areas of CO_2_ and APs emissions extracted from gridded maps (i.e., CEDS, EDGAR, PKU, MEIC, and REAS) during 2010–2015. The CO_2_, CO, and other APs emissions are in units of 10^3^ Mt yr^−1^, 10 Mt yr^−1^, and Mt yr^−1^, respectively

The five available gridded emissions inventories of CEDS, EDGAR, MEIC, PKU, and REAS were analyzed to explore the spatial characteristics of CO_2_ and APs emissions. Regions with high anthropogenic emissions generally host a large population and rapid economic and industrialized development. Therefore, seven high-emitting areas were analyzed to identify the major sources and possible linkages between pollutants and CO_2_ emissions via multiple inventories (Fig. 3). During 2010–2015, the BTHs and YRD regions were the main contributors to the national total emissions (Fig. 3d, e). The ensemble mean CO_2_ emissions from the BTHs and YRD regions contributed 21.9% and 16.6%, respectively, to the total emissions, while the emissions in the PRD region only contributed 2.7%. Given the great demand for energy and motorization in metropolitan areas, high carbon emissions are often accompanied by high emissions of APs. For example, the highest APs emissions were generally located in the BTHs and YRD regions, accounting for 19.5%–23.7% and 9.0%–16.8%, respectively, of the total APs emissions. The CY, FP, NE, and TC regions contributed approximately 3.5%–10.2% to the total emissions, while the PRD region only contributed 1.1%–4.0%. Specifically, nearly all of the CO_2_ high-emitting areas attained relatively high emissions level of CO, NO_x_, NMVOC, and SO_2_. These close linkages occur because these emissions are largely contributed by power and industrial sources (Fig. 2). Because of the common sources, control measures targeting specific APs should be considered complementary to CO_2_ mitigation strategies [14, 24]. The consistent spatial patterns of pollutants and CO_2_ emissions reveal the importance of synergy in controlling emissions in high-emitting areas and setting source-specific emissions reduction targets.

**Fig. 4:**
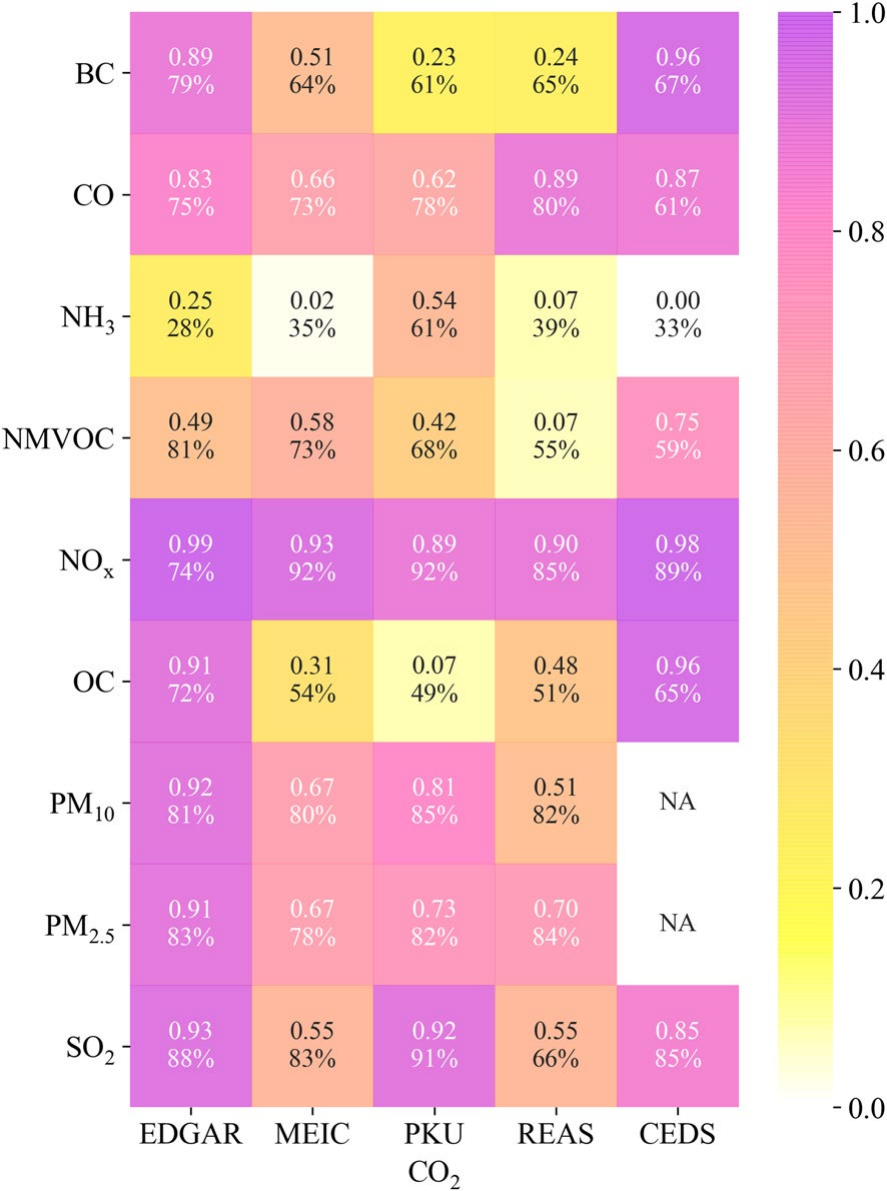
Correlation relationships between CO_2_ and APs emissions in the top 5% high-emitting grids. The numbers within each grid represent the coefficient of determination (R^2^) and the percent number is the common grid cells of CO_2_ and APs compared to the top 5% CO_2_ emissions grids. NA denotes not available

To further identify the relationship between CO_2_ and APs emissions at the grid cell level, the numbers of the top 5% high-emitting grids (representing emissions hotspots) were extracted from CO_2_ inventories to detect the consistency and difference between CO_2_ and APs emissions. As shown in Fig. 4, the highest correlation was found between CO_2_ and NO_x_, with the coefficient of determination (R^2^) mostly remaining above 0.9, and the numbers of common grid cells of CO_2_ and APs hotspots reached more than 70% of all grid cells during 2010–2015 (Fig. 4). Strong correlations were observed between CO_2_ and CO, SO_2_, and PM emissions, with R^2^ values generally exceeding 0.6, and the common grid cells accounted for more than 60% of the total number of grid cells. We further explored the spatial distributions of such high emission grid cells, and found the co-exist of high emissions for both CO_2_ and fossil APs (Fig. S6). For example, we found the emissions hotspots (big bubbles in (Fig. S6) in the Beijing-Tianjin-Hebei regions and the Yangtze River Delta region. These results revealed that there occurred highly consistent patterns in source locations and high-emission intensity areas. These results were probably due to that the power and industrial sources were the dominant contributors (Fig. 2). There appeared to be a limited correlation between CO_2_ and BC or OC based on MEIC, PKU, and REAS, with R^2^ values ranging from 0.1 to 0.5. This is because the dominant contributor to BC and OC emissions was the residential sector. Residential emissions were from buildings and spatial randomly distributed, and thus they were not always consistent with high emissions of fossil CO_2_ (power and industry). However, a strong correlation was observed between CO_2_ and BC or OC based on EDGAR and CEDS. This could be attributed to the power and industrial sources largely contributing to BC and OC in EDGAR. Similarly, the relationship between CO_2_ and NMVOC emissions was influenced by the different emission sources. Solvent use and industry dominated the growth in NMVOC emissions (Li 2019), while CO_2_ was mainly driven by the power and industrial sectors. Hence, the R^2^ values generally ranged from 0.42 to 0.75. NH_3_ emissions hotspots indicated a weak relationship with CO_2_ (R^2^ < 0.3) and the smallest number of common grids (28%–39%). The agriculture sector was the major contributor to NH_3_ emissions (Fig. 2a, h). The relatively higher positive correlation (R^2^ = 0.54) between NH_3_ and CO_2_ based on PKU was largely because PKU mainly considered emissions from combustion and industrial sources but did not include agriculture [61].

### Relationships between the changes in CO_2_ and APs emissions over time

**Fig. 5:**
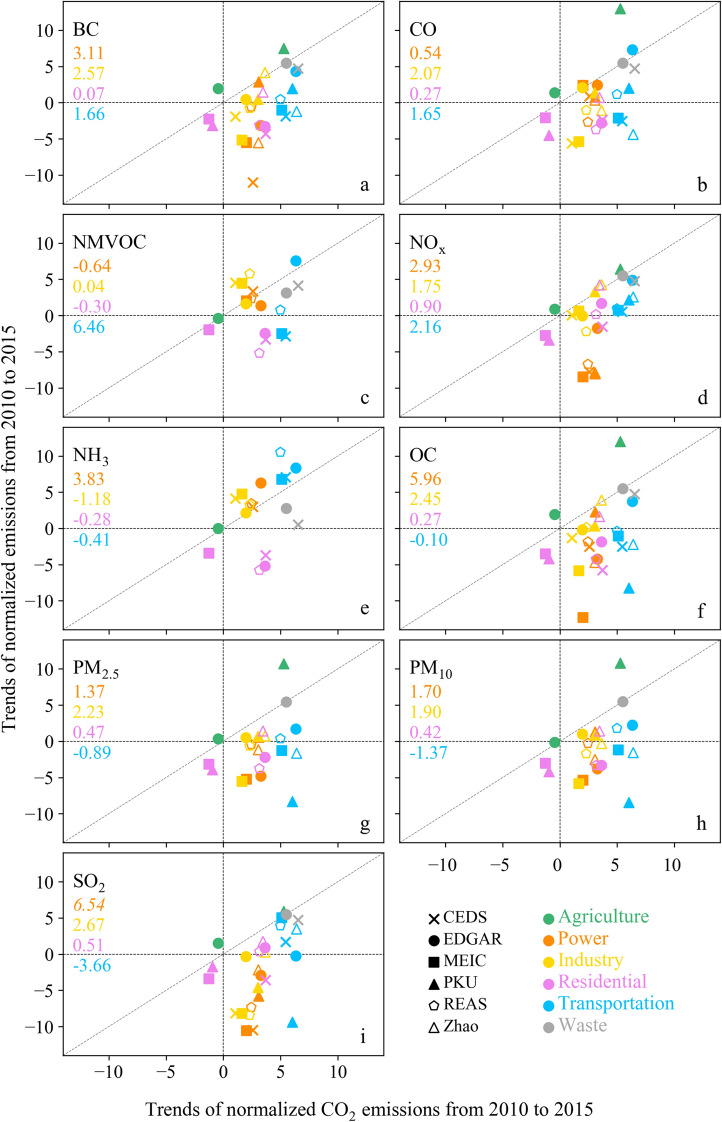
Relationships between the changes in CO_2_ and APs emissions over time among the inventories during 2010–2015. The numbers represent the slopes of the linear regression lines, and the italic numbers indicate the statistical significance (*P* < 0.05)

China’s emissions have dramatically changed, especially after the implementation of control targets for the emissions of specific APs and carbon emissions in recent years. To quantify the relationships between CO_2_ and APs emissions changes over time, 6 inventories, including CEDS, EDGAR, MEIC, PKU, REAS, and Zhao, were evaluated at the sectoral level during 2010–2015. As illustrated in Fig. 5, in the residential sector, effective controls of APs and CO_2_ emissions were observed based on MEIC and PKU, which were located in the third quadrant. In the power and industrial sectors, pollution generally exhibited negative changes with CO_2_ emissions growth, which was mainly located in the fourth quadrant (Fig. 5c, d, f, h–k). This further confirmed that actions to mitigate air pollution were more effective than was limiting CO_2_ emissions. However, for NMVOC and NH_3_, emissions still increased with CO_2_, which was located in the first quadrant. This could be because NMVOC and NH_3_ emissions were mainly driven by persistent growth due to the industry and solvent use and the lack of relevant emissions controls over SO_2_, NO_x_, and PM [45, 46, 70].

Pollutants emissions stemming from the waste sector tended to increase with CO_2_ among the inventories. The total amount of municipal solid waste continues to grow with the population, urbanization, and industrialization levels [80]. The amount of solid waste treatment increased by 25% during 2010–2015, and the level consistently increased to 10.11 Mt in 2019 [81]. Although the power and industrial sectors are the dominant emission sources in China, transportation has contributed a growing share to the total emissions due to the increase in motorization (Fig. S2). In the transportation sector, pollutants emissions based on PKU, MEIC, and Zhao reflected negative changes with CO_2_ growth (in the fourth quadrant, as shown in Fig. 5a, b, f–h), except for NO_x_ and NH_3_ (in the first quadrant, as shown in Fig. 5d, e). In the agriculture sector, emissions indicated low interannual variability among the inventories, except for PKU. This discrepancy was mainly attributed to PKU only including the enhanced contributions of both fossil fuel and biomass combustion to the agricultural emissions.

After the implementation of stringent air quality control measures for several years after 2013, the majority of pollutants exhibited a decreasing trend. Moreover, compared to the 2000s, the growth in CO_2_ emissions has successfully declined in recent years (Fig. S4). During 2000–2009, APs emissions were positively correlated with CO_2_, and the trends were generally located in the first quadrant (Fig. S4). It is encouraging to find that China’s efforts to mitigate both air pollution and climate change have taken effect. To further reduce GHGs and APs emissions, more effective strategies are needed to strengthen controls on NMVOC and NH_3_ emissions and emissions originating from vehicle and waste sources.

## Conclusions

Driven by the increase in energy consumption, urbanization, and vehicle number, air pollution and carbon emissions in China have increasingly become a serious problem, especially due to their negative impacts on air quality, human health, and climate change. Knowledge of spatiotemporal characteristics and exploration of possible links between APs and GHGs are imperative to effectively mitigate both air pollution and climate change. Through analysis of six global and regional bottom-up inventories, the results in this study revealed that CO_2_, NO_x_, and SO_2_ emissions were closely linked because they were all mainly driven by the power and industrial sectors during 1980–2015. In regard to PM_10_, PM_2.5_, CO, BC, and OC, the residential and industrial sectors were the largest contributors to the total emissions. Both APs and GHGs exhibited a decreasing emissions share stemming from residential consumption, especially for CO, BC, and NMVOC, with the proportions decreasing more than 30%. However, the transportation sector increased its impact on recent emissions, particularly in NO_x_ and NMVOC emissions. After the implementation of strict pollution control policies in 2013, along with technological improvements, the emissions of most APs and CO_2_ were reduced or negative growth was accomplished in the power and industrial sectors. Spatially, seven high-emitting areas all indicated high-level emissions of CO, NO_x_, SO_2_, and NMVOC in line with CO_2_ emissions. Moreover, strong correlation relationships (R^2^ > 0.6) were found between CO_2_ and PM, CO, NO_x_, CO, and SO_2_ emissions in the top 5% high-emitting grid cells during 2010–2015. The emissions of major APs and CO_2_ revealed highly consistent patterns in terms of source allocation and emission intensity. We acknowledge that this study did not examine underlying inventory errors and uncertainties due to lack of more detailed data. To further reduce pollutants and carbon emissions, ongoing efforts are needed to coherently consider the main emissions sectors and trends of APs and CO_2_ emissions and their linkages to simultaneously address climate change and air pollution problems.

## Supplementary Information

**Additional file 1.** Additional figures and tables.

## Data Availability

The data and materials used in this paper are provided in the supplement. And data and materials are also available at https://doi.org/10.6084/m9.figshare.16614016.v3.
