# Supplementary material for: An integrated view of correlated emissions of greenhouse gases and air pollutants in China
Source: Carbon Balance Manag. 2023 May 19;18:9. doi: 10.1186/s13021-023-00229-x (PMC10199610; doi:10.1186/s13021-023-00229-x)
Supplement: Supplementary file 1 — Additional file 1. Additional figures and tables. [file 13021_2023_229_MOESM1_ESM.docx]

*Supplementary file for:*

An integrated view of correlated emissions of greenhouse gases and air pollutants in China

Xiaohui Lin^1^, Ruqi Yang^2^, Wen Zhang^1*^, Ning Zeng^3^, Yu Zhao^4^, Guocheng Wang^1^, Tingting Li^1,5^，Qixiang Cai^2*^

^1^State Key Laboratory of Atmospheric Boundary Layer Physics and Atmospheric Chemistry, Institute of Atmospheric Physics, Chinese Academy of Sciences, Beijing, China

^2^State Key Laboratory of Numerical Modeling for Atmospheric Sciences and Geophysical Fluid Dynamics, Institute of Atmospheric Physics, Chinese Academy of Sciences, Beijing, China

^3^Department of Atmospheric and Oceanic Science, and Earth System Science Interdisciplinary Center, University of Maryland, College Park, Maryland, USA

^4^State Key Laboratory of Pollution Control & Resource Reuse and School of the Environment, Nanjing University, 163 Xianlin Ave., Nanjing, Jiangsu, China

^5^Southern Marine Science and Engineering Guangdong Laboratory (Zhuhai), Zhuhai, Guangdong, China

^*^ *Correspondence to*: [zhw@mail.iap.ac.cn](mailto:zhw@mail.iap.ac.cn); [caiqixiang@mail.iap.ac.cn](mailto:caiqixiang@mail.iap.ac.cn).


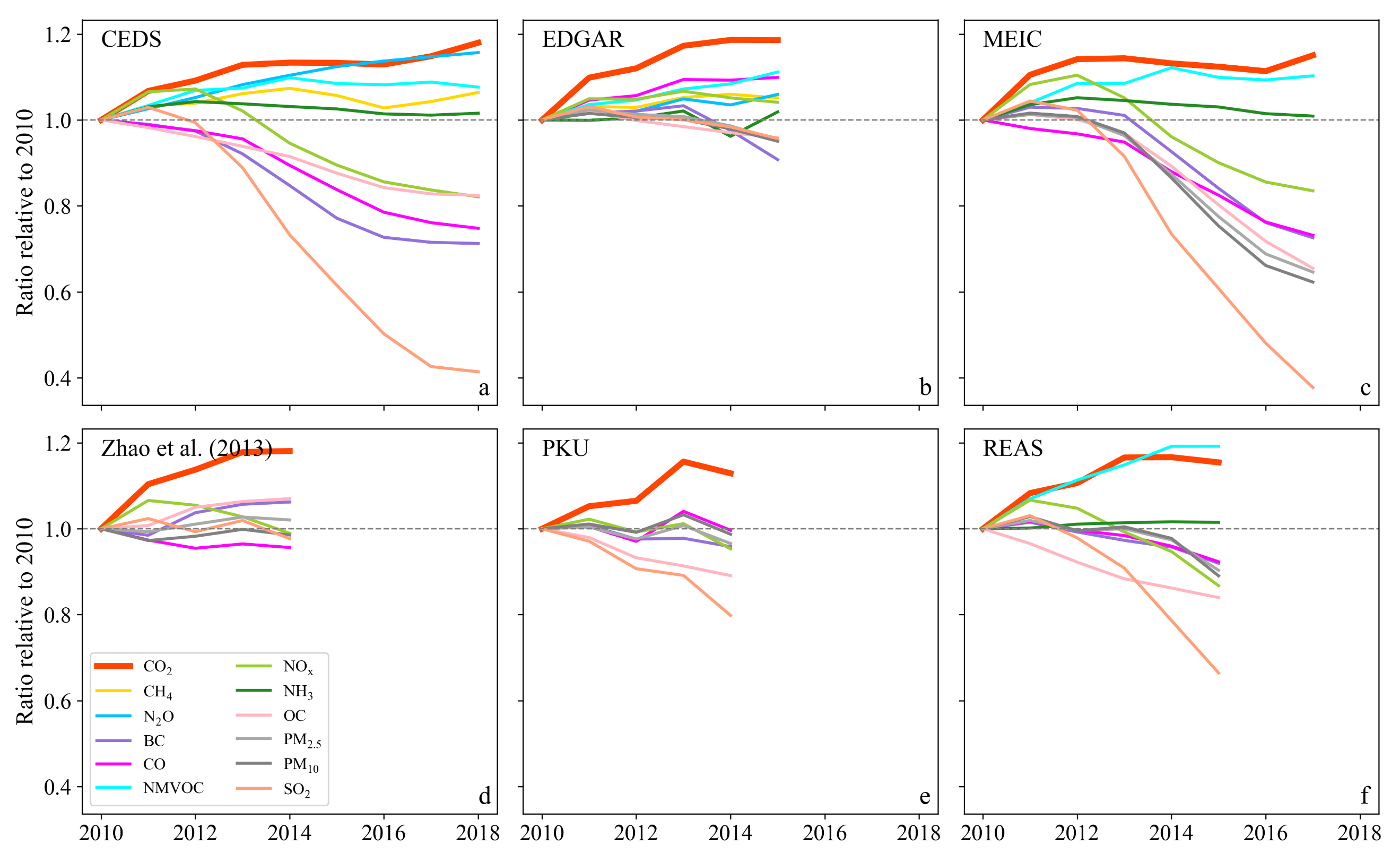


Fig. S1 Trends of GHG and APs emissions during 2010-2018. Data are normalized by dividing the value of each year by their corresponding values in 2010.


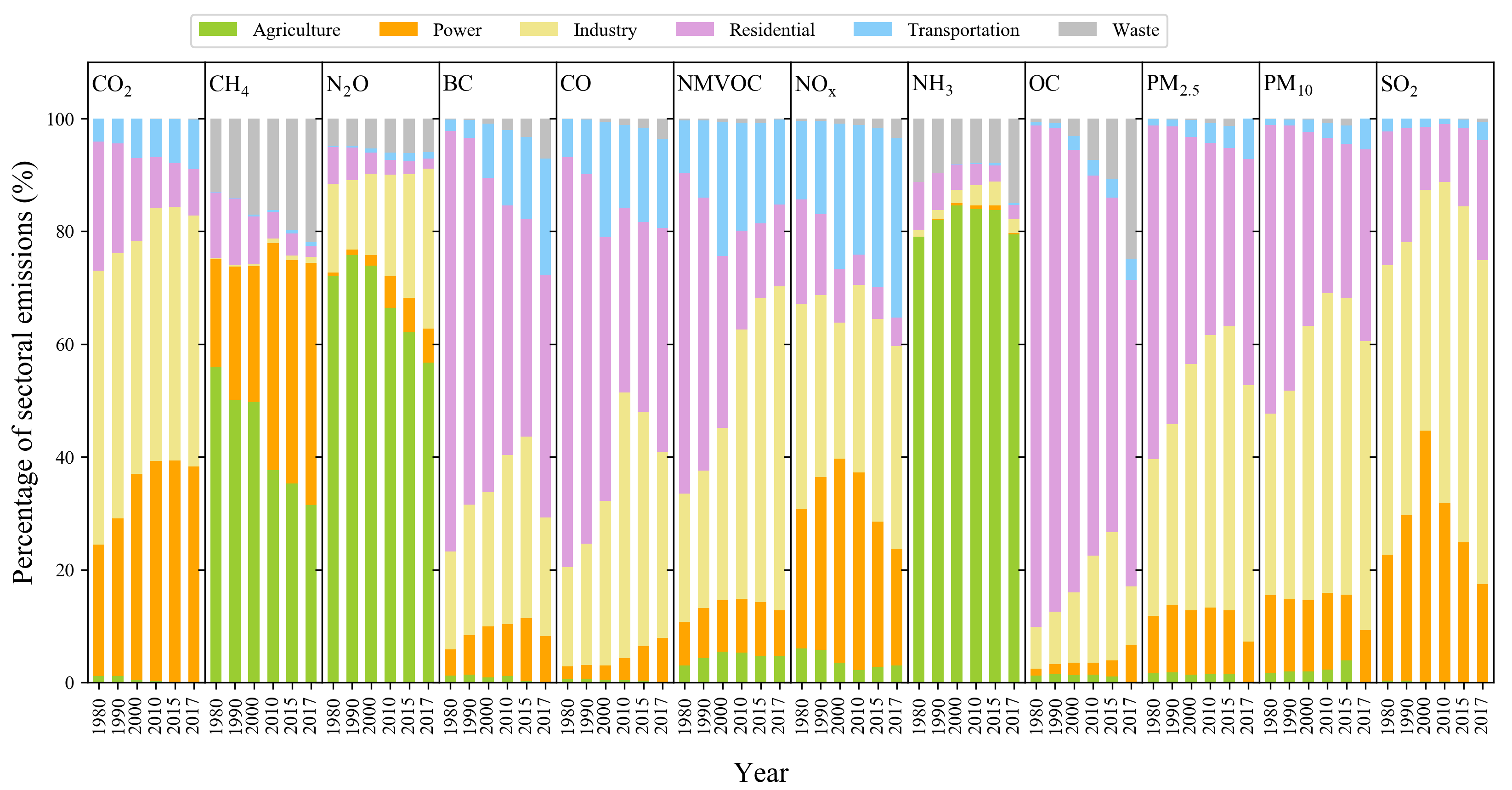


Fig. S2 Sectoral contributions to China’s GHG and APs emissions and their variations during 1980-2017.


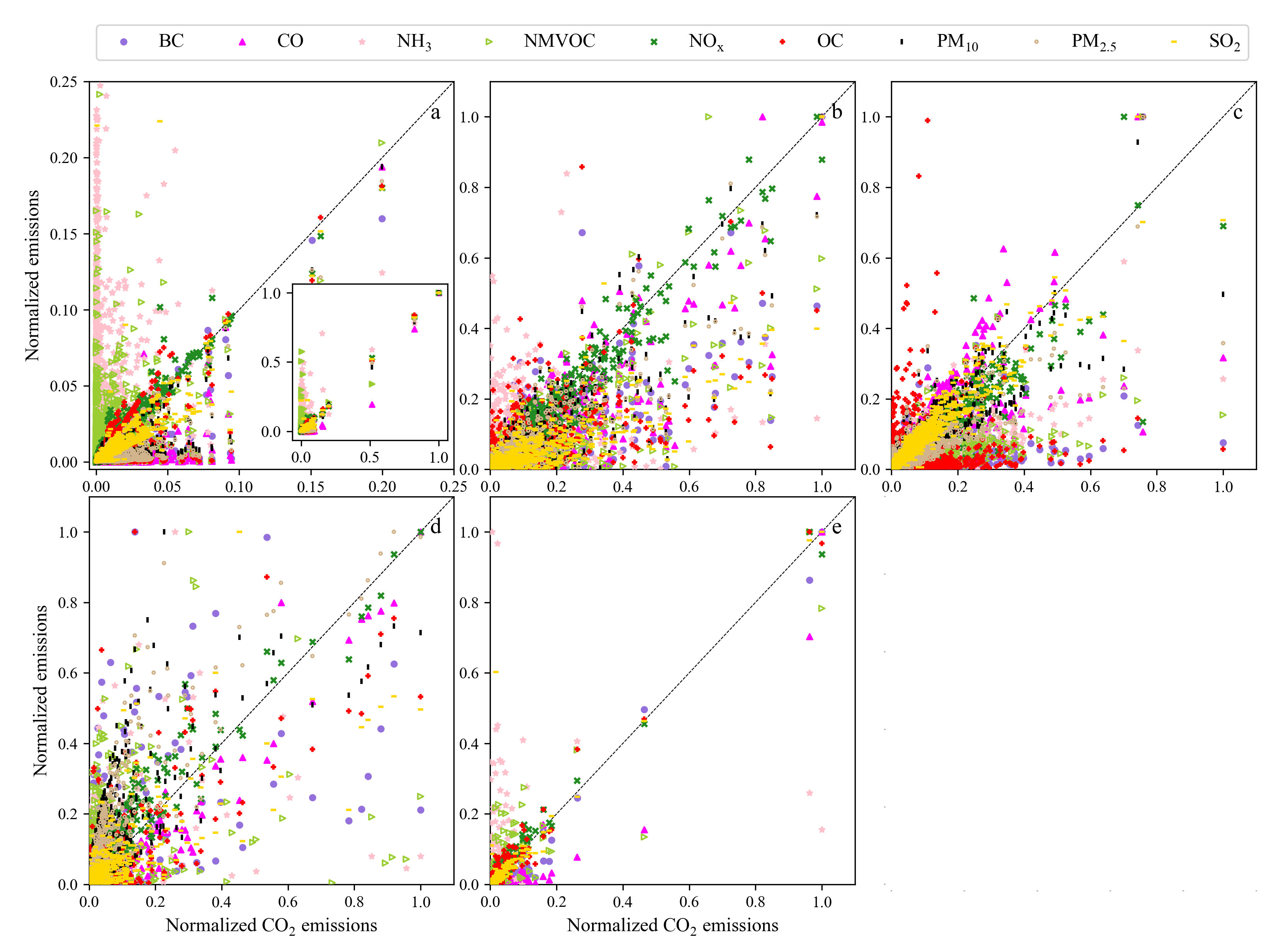


Fig. S3 The relationships between CO_2_ and APs emissions in the top 5% high-emitting grids during 2010-2015. Subplots (a) to (e) denote results from inventory of EDGAR, MEIC, PKU, REAS and CEDS. Inset map in subplot (a) shows a full x-y range for normalized emissions, and the outset map shows the zoomed in view of inset map.


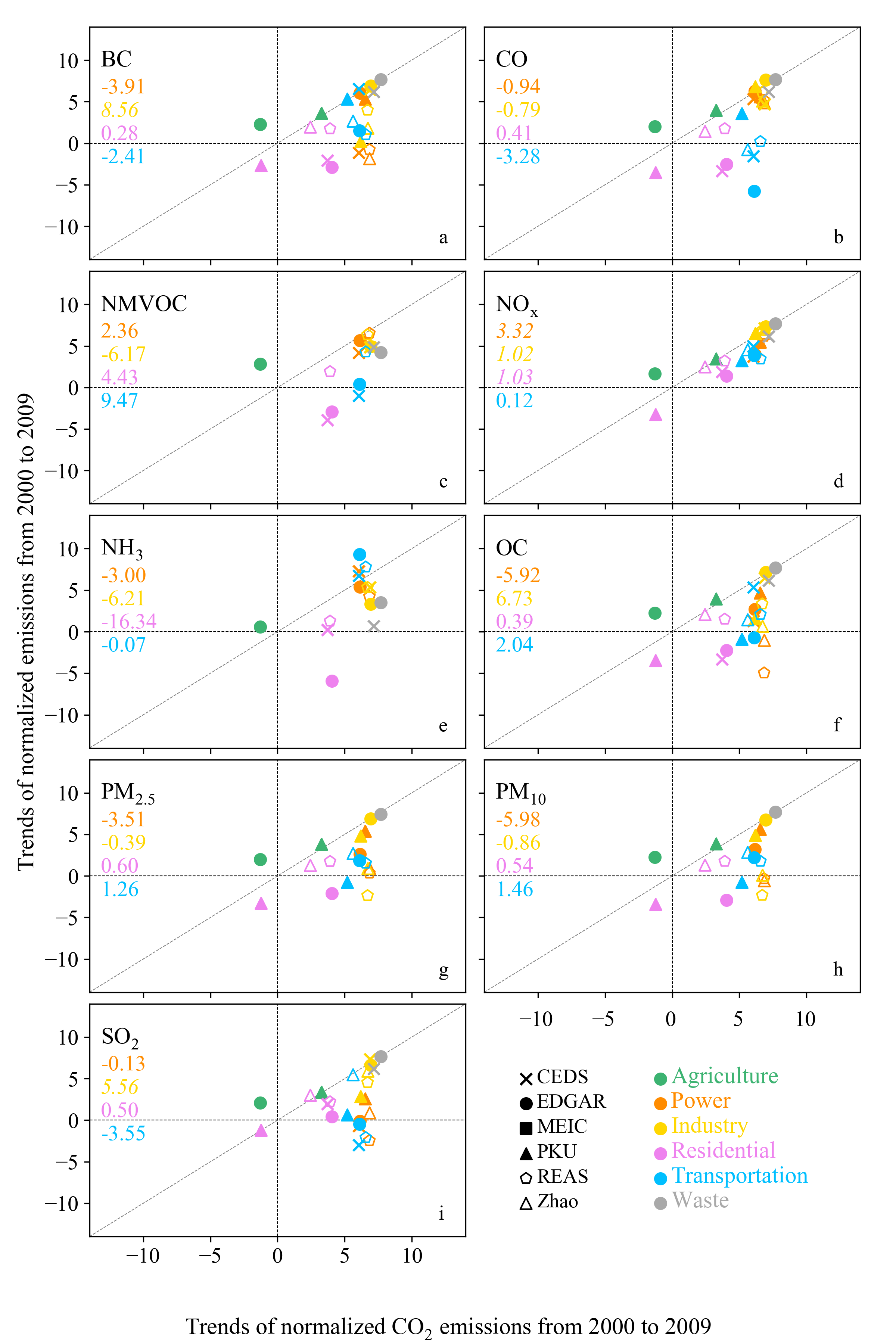


Fig. S4 The relationships of changes in CO_2_ and APs emissions over time among inventories during 2000-2009. The numbers represent the slopes of linearly regression and italic numbers indicate statistically significant (*P*< 0.05).


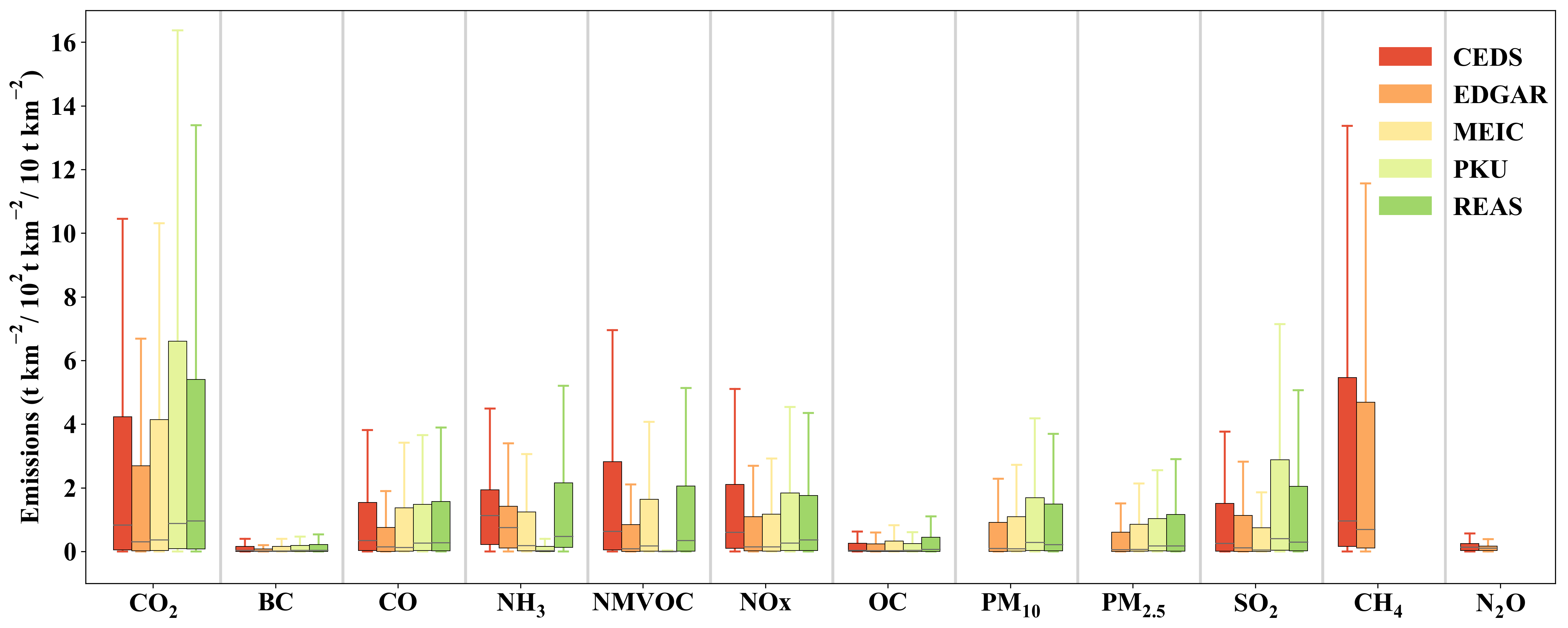


Fig. S5 Boxplots of the GHGs and APs emissions for all grid cells during 2010-2015.


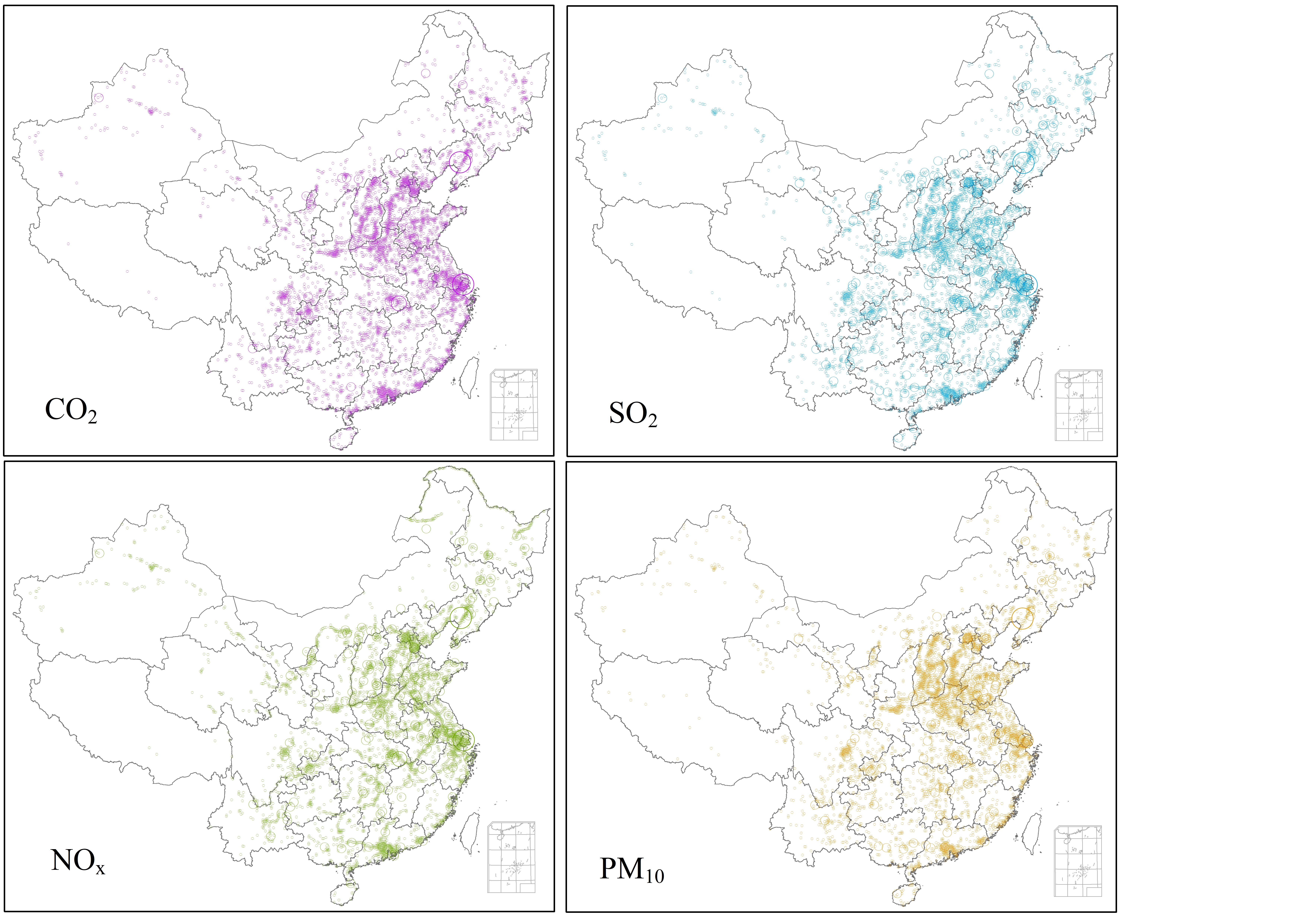


Fig. S6 Bubble plots of the emissions of CO_2_, SO_2_, NO_x_ and PM_10_ for top 5% grid cells for the average of 2010-2015 in EDGAR.

| Table S1 Differences and uncertainties (%) of GHGs and APs emissions in China*. | | | | | | | | | | | | | |
| --- | --- | --- | --- | --- | --- | --- | --- | --- | --- | --- | --- | --- | --- |
| Data | Year | CO2 | CH4 | BC | CO | NH3 | NMVOC | NOx | OC | PM10 | PM2.5 | SO2 | Reference |
| REAS | 2015 | ±19 |  | ±111 | ±73 | ±82 | ±76 |  | ±193 | ±83 | ±94 | ±40 | [[1](#_ENREF_1)] |
| Zhao | 2010 | (-10,9) |  | (-28,126) | (-18,42) |  |  |  | (-42,114) | (-15,54) | (-15,63) | (-15,26) | [[2](#_ENREF_2)] |
| MEIC | 2006 |  |  |  | ±70 |  | ±68 | ±31 |  | ±132 | ±130 | ±12 | [[3](#_ENREF_3)] |
| EDGAR | 2012 | 9 | 57 | 74.3 | 94.4 | 294.4 | 138.6 | 56.2 | 129.2 | 81.7 | 75.7 | 22.2 | [[4](#_ENREF_4), [5](#_ENREF_5)] |
| *The uncertainty of CO2 and CH4 in EDGAR was based on 2 standard deviations (std.), and others were 1 std. | | | | | | | | | | | | | |

References

1. Kurokawa J, Ohara T. Long-term historical trends in air pollutant emissions in Asia: Regional Emission inventory in ASia (REAS) version 3. Atmospheric Chemistry and Physics. 2020;20(21):12761-93.

2. Zhao Y, Zhang J, Nielsen C. The effects of recent control policies on trends in emissions of anthropogenic atmospheric pollutants and CO 2 in China. Atmospheric Chemistry and Physics. 2013;13(2):487-508.

3. Zhang Q, Streets DG, Carmichael GR, He KB, Huo H, Kannari A, et al. Asian emissions in 2006 for the NASA INTEX-B mission. Atmos Chem Phys. 2009;9(14):5131-53.

4. Janssens-Maenhout G, Crippa M, Guizzardi D, Muntean M, Schaaf E, Dentener F, et al. EDGAR v4.3.2 Global Atlas of the three major greenhouse gas emissions for the period 1970–2012. Earth Syst Sci Data. 2019;11(3):959-1002.

5. Crippa M, Guizzardi D, Muntean M, Schaaf E, Dentener F, van Aardenne JA, et al. Gridded emissions of air pollutants for the period 1970–2012 within EDGAR v4.3.2. Earth Syst Sci Data. 2018;10(4):1987-2013.
